# T141. CHARACTERIZATION OF HEMODYNAMIC ALTERATIONS IN SCHIZOPHRENIA AND BIPOLAR DISORDER AND THEIR EFFECT ON RESTING-STATE FUNCTIONAL CONNECTIVITY

**DOI:** 10.1093/schbul/sby016.417

**Published:** 2018-04-01

**Authors:** Wenjing Yan, Lena Palaniyappan, Peter Liddle, Gopikrishna Deshpande

**Affiliations:** 1 Auburn University; 2 University of Western Ontario; 3 University of Nottingham

## Abstract

**Background:**

Schizophrenia (SZ) and bipolar disorder (BP) have both common and distinct clinical symptomatology. Their neural bases have been explored using functional connectivity between brain regions using resting-state functional magnetic resonance imaging (rs-fMRI). However, fMRI is an indirect measure of neural activity and is modeled as a convolution of the hemodynamic response function (HRF) and latent neural activity. The HRF varies across both individuals and different brain regions within an individual. Consequently, it is plausible for two brain regions to appear synchronized in the BOLD space while being desynchronized in latent neural space and vice versa.

**Methods:**

In order to address this issue, we estimated voxel-specific HRFs by deconvolving rs-fMRI time series obtained from SZ (N=19), BP (N=35) and matched healthy individuals (N=34). The shape of the HRF was significantly different between the three groups in many regions previously implicated in SZ and BP. Specifically, we found voxels within the mediodorsal, habenular and central lateral nuclei of the thalamus to have HRFs with aberrations in all three of its shape parameters: time to peak, response height and full width half max. Therefore, we defined this region as the seed, estimated seed-based functional connectivity maps in all three groups and characterized pairwise differences between them. Further, we performed a 2-way ANOVA and estimated regions exhibiting an interaction between the group and deconvolution factors.

**Results:**

We found voxels within the mediodorsal, habenular and central lateral nuclei of the thalamus to have HRFs with aberrations in all three of its shape parameters: time to peak, response height and full width half max. Results indicated that functional connectivity differences between the groups are inferred significantly differently with raw BOLD and deconvolved latent neural time series. Since the variability of the HRF could be driven by both neural and non-neural factors, we feel that it is preferable to estimate functional connectivity using deconvolved data.

**Discussion:**

Neurochemicals such as GABA, glutamate, serotonin and nitric oxide have a role in controlling neurosignaling pathways underlying neurovascular coupling and hence the HRF. Previously documented alterations of these neurochemicals in SZ and BP could, at least in part, explain the significant differences in HRF shapes observed between the groups. Functional connectivity group differences obtained from raw BOLD data must be interpreted cautiously in the light of systematic HRF differences between groups.

